# Integration of Transformer-Based Architecture and Large Language Models for Optical Coherence Tomography Data Analysis to Improve the Accuracy of Differential Diagnosis of Retinal Diseases

**DOI:** 10.17691/stm2026.18.3.01

**Published:** 2026-06-30

**Authors:** O.V. Konshina, A.D. Pershin, M.K. Kulyabin, V.I. Borisov

**Affiliations:** 1 Master Student, Educational and Research Center “Artificial Intelligence”; Ural Federal University named after the First President of Russia B.N. Yeltsin, Institute of Radioelectronics and Information Technologies — RTF, 32 Mira St., Ekaterinburg, 620062, Russia; Ophthalmologist; MIK Clinic, 21 Korablestroiteley St., Bldg. 1, Block B, Saint Petersburg, 199226, Russia; 2 PhD Student, Educational and Research Center “Artificial Intelligence”; Ural Federal University named after the First President of Russia B.N. Yeltsin, Institute of Radioelectronics and Information Technologies — RTF, 32 Mira St., Ekaterinburg, 620062, Russia; 3 PhD, Technical Director; LLC “VisioMedAI”, 8 Golovinskoye Shosse, Moscow, 125212, Russia; 4 PhD, Associate Professor, Educational and Research Center “Artificial Intelligence”; Ural Federal University named after the First President of Russia B.N. Yeltsin, Institute of Radioelectronics and Information Technologies — RTF, 32 Mira St., Ekaterinburg, 620062, Russia

**Keywords:** OCT biomarker classification, multiclass OCT classification, LLM in ophthalmology, deep learning, retina, ViT, DeepSeek

## Abstract

**The aim of the present study** was to improve the differential diagnosis accuracy of retinal diseases combining a transformer model to classify the biomarkers on OCT images and the large language model DeepSeek-V3.

**Materials and Methods:**

Two datasets were collected and annotated: a training set (3288 central retinal OCT images annotated for 8 biomarkers) and a validation set (50 clinical cases from octcases.com). For biomarker classification, we compared ResNet, DenseNet, EfficientNet, and Vision Transformer (ViT-Tiny-Patch16-224) architectures. The ViT-Tiny model demonstrated the highest performance, its F1 macro — 0.84±0.03. An integration algorithm was developed to combine predicted biomarker labels with clinical history data via the DeepSeek-V3 LLM API.

**Results:**

Combining OCT biomarkers and the medical history significantly improved the diagnostic accuracy: Top-1 Accuracy — 78%, Top-3 Accuracy — 94%, MRR — 84%, representing 10–44% improvement over using either data type alone.

**Conclusion:**

The suggested approach enabled to automate the detection of biomarkers on OCT images and enhance the differential diagnosis accuracy of eye diseases, reducing the image interpretation time and supporting clinical decision-making.

## Introduction

Currently, it is being studied the feasibility of implementing modern technological decisions, particularly, artificial intelligence systems, into medical practice [1–3]. Increasingly greater attention is given to the application of large language models (LLM), such as GPT, Gemini, Grok, and DeepSeek [4–7]. The models have demonstrated high efficacy in the natural language processing, generating medical conclusions, and the analysis of complicated case histories. Nevertheless, their integration with imaging data requires further research that is particularly urgent within the context of using optical coherence tomography (OCT). OCT is a noninvasive imaging technique, which enables to make a high-precision assessment of retinal structures at the cellular level, the resolution being up to 2–3 μm [8], and detect over 40 different biomarkers typical for a wide range of retinal diseases [9]. In scientific literature there are numerous studies devoted to the classification of retinal disorders based on OCT images using machine and deep learning methods [10–16]. However, most researchers are restricted by the classification aiming at four diseases, whereas in real clinical settings the number of potential diagnoses significantly exceeds this number. An alternative approach consists in revealing and classifying the biomarkers on OCT images [17–20]. In this respect, it should be considered that one and the same biomarker can occur in different diseases, and one diagnosis can be presented by different sets of biomarkers. Therefore, to improve the differential diagnosis accuracy, it is necessary to consider both: imaging data and the patient’s past history.

**The aim of the present study** was to improve the accuracy of the differential diagnosis of retinal diseases combining a transformer model to classify the biomarkers on OCT images and the large language model DeepSeek-V3.

## Materials and Methods

### Datasets

The study involved two datasets. A model training set for a multiclass classification was formed from three sources: open datasets: OCTID (946 images) [21] and OCTDL (2042 images) [22], as well as a personal dataset (300 OCT macular images). The summary dataset included 3288 images annotated for eight biomarkers: IRF — intraretinal fluid; SRF — subretinal fluid; HE — hard exudates; CNV — choroidal neovascular membranes of all types + subretinal hyperreflective material; PED — pigment epithelium elevation; Drusen — all drusen types; ERM — epiretinal membrane; MH (macular hole) and LMH (lamellar macular hole) — the union of all types of macular holes.

**Figure 1.**
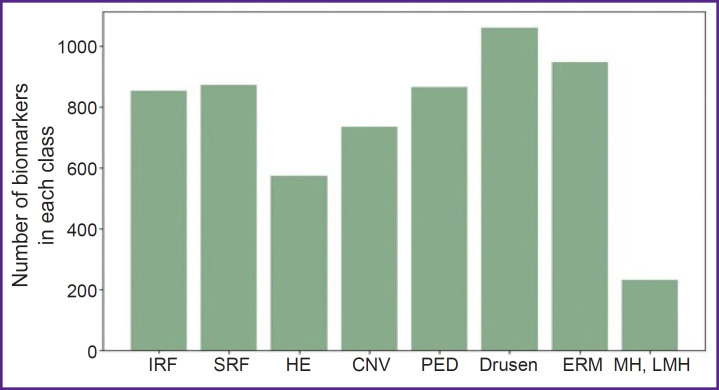
Biomarker class distribution histogram

Figure 1 represents the distribution of biomarkers in a training set. The dataset preserves the imbalance of classes. Each image can contain several biomarkers.

As a test dataset we used the annotated clinical cases from an open professional OCT site — octcases. com. The dataset contained the following data: a medical history, an OCT image and a verified diagnosis. There were altogether 18 different diagnoses represented in a dataset, some of them were rare ones.

The images were preliminarily processed using torchvision library, and there was a succession of transformations: scaling, random image rotations and flipping, exploded and centered cropping and autopadding, transformation into tensors and normalization. These stages provided the image standardization and augmentation to improve the model training quality.

### The best model training and selection for the multiclass biomarker classification on retinal OCT images

There was formed a set of models to solve the tasks of a multiclass classification and study the effect of different architectures on the model performance: ResNet50 and ResNet101 (convolutional neural networks with different depth), DenseNet121 (the convolutional neural network with dense links between the layers), EfficientNet_b1 (the convolutional neural network with effective scaling), and ViT-Tiny-Patch16-224 (Vision Transformer; transformer architecture). It provided the diversity of approaches and enabled to assess the feasibility of the models to the task in limited training data volume.

The following multiclass classification metrics were calculated for each model (macro averaging — the metrics averaging by classes with equal weight of each class; micro averaging — the calculation of metrics based on the sum of true-positive, false-positive and false-negative predictions by all classes, it enables to consider the contribution of each class in proportion to the number of objects in the class):

Accuracy — the proportion of correctly classified objects among all those observed;
Precision — the proportion of true-positive predictions among all positive objects predicted by the model;
Recall — the proportion of true-positive objects correctly detected by the model among all true-positive ones;
F1-score — harmonic mean between Precision and Recall characterizing the balance of precision and recall.

95% confidence intervals were formed for each metric on a test dataset using a bootstrap method. Table 1 demonstrates the obtained quality metrics of the trained models for the multiclass classification of biomarkers on retinal OCT images, the metrics being calculated on a test dataset with confidence intervals.

**Table 1.** Pre-trained model quality metrics for the multiclass classification of biomarkers on retinal OCT images

Model	F1 micro	F1 macro
ResNet50	0.81±0.02	0.81±0.02
ResNet101	0.80±0.03	0.80±0.03
DenseNet121	0.75±0.04	0.76±0.04
EfficientNet_b1	0.64±0.04	0.61±0.04
**ViT-Tiny**	**0.84±0.02**	**0.84±0.03**

The best result in the task was demonstrated by ViT-Tiny-Patch16-224 with transformer architecture. A weighted F1-score for the model was 0.84±0.04; Specificity — 0.94±0.01. It was the model to be further used for the classification validation and the interaction with a large language model.

### Validation and interaction with a large language model

**Figure 2.**
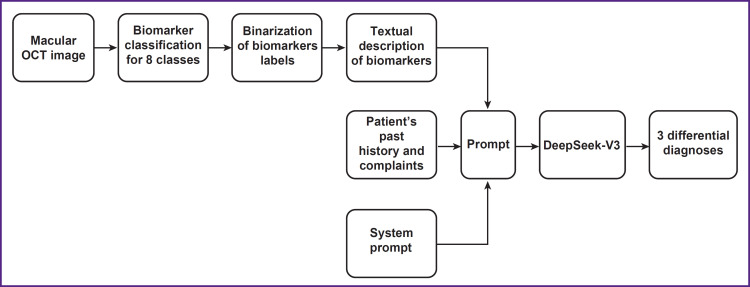
The integration flow chart of the transformer model ViT-Tiny and the large language model DeepSeek-V3

Figure 2 represents the architecture of the suggested approach.

There was formed a prompt (a language model request) from the labels of the biomarkers of diseases and past history data using a trained model ViT-Tiny-Patch16-224 to interact with LLM DeepSeek-V3 through the application programmer interface (API). The model exit labels were binary for the multiclass classification, and converted into the textual description of classes. Three prompt variants were formed to assess the algorithm operation:

1. OCT image description and medical history data;
2. the description of OCT images alone;
3. only the medical history data.

The following metrics were used to assess the algorithm operation:

Top-1 Accuracy — the proportion of cases, when the correct class (in our situation: the correct diagnosis) was in the first position;
Top-3 Accuracy — the proportion of cases, when the true class (in our situation: the true diagnosis) appeared to be among three most probable predictions of the model;
MRR — the mean reciprocal rank indicating where on average there is the correct answer among all predictions.

## Results

The ViT-Tiny-Patch16-224 model was evaluated using Grad-CAM++. Grad-CAM++ (Gradient-weighted Class Activation Mapping) is the technique applied to interpret the deep learning models, which enables to visualize significant image areas used by the model for decision making [23].

**Figure 3.**
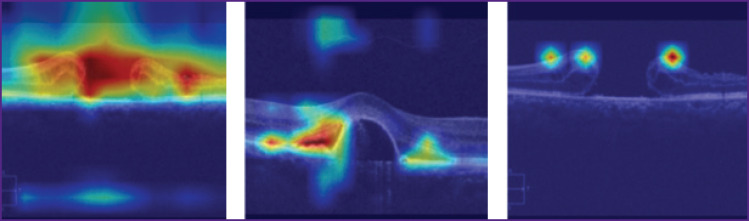
Activation cards for different biomarker classes (from left to right — MH, SRF, ERM classes)

Figure 3 represents the examples of activation cards for different biomarker classes. The model is focused on certain image areas that can be useful to understand how the model makes decisions.

Table 2 demonstrates the interaction algorithm assessment of pre-trained model ViT-Tiny-Patch16-224 and LLM DeepSeek-V3 in the differential diagnostics of retinal diseases.

**Table 2.** LLM DeepSeek-V3 operation quality metrics with different prompt types

Metric	Prompt		
	OCT + past history	OCT	Past history
Top-1 Accuracy	0.78±0.11	0.52±0.14	0.30±0.14
Top-3 Accuracy	0.94±0.08	0.66±0.13	0.46±0.14
MRR	0.84±0.04	0.58±0.06	0.41±0.06

Table 2 shows the probability of correct diagnosis among three most probable variants (Top-3 metric) to be increasingly growing when passing from the use of medical history data only (46%) to the analysis of OCT images alone (66%), and reaching its maximum when using the combined architecture uniting the medical history and OCT data (94%). According to the findings, the following conclusions can be made: the best results can be achieved when using the combined data obtained using the neural network Vit-Tiny (pre-trained aimed at the multiclass classification of biomarkers on OCT images) and medical history textual data.

Table 3 represents the examples and the practical aspects of the suggested approach applicability (the algorithm operation with different prompt types).

**Table 3.** The algorithm operation examples with different prompt variants

OCT macular images	Prompt variants	Algorithm output (3 differential diagnoses)
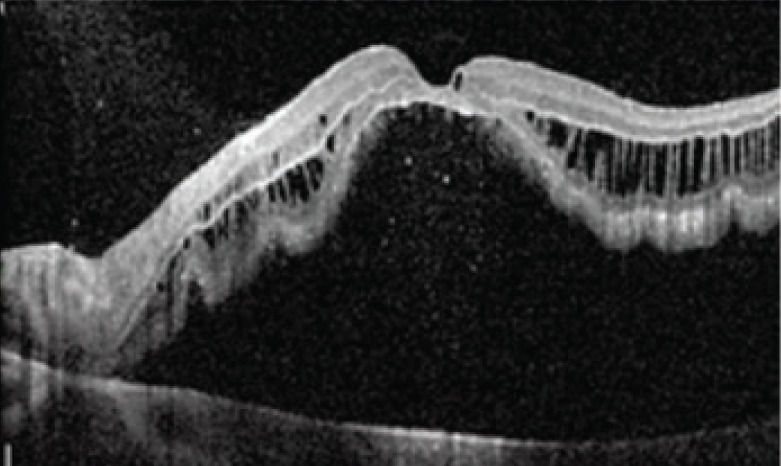 Verified diagnosis: **“retinal detachment”** The biomarkers labeled by the transformer model: **IRF, SRF**	***OCT*** You are an ophthalmologist. Clinical case study: on an OCT image of the macular area, there are revealed IRF, SRF Describe briefly 3 most probable diagnoses. Only the titles of clinical diagnoses	1. Age-related macular degeneration 2. Central serous chorioretinopathy 3. Diabetic macular edema
	***Past history*** You are an ophthalmologist. Clinical case study: an 11-year-old boy with aphakia and microspherophakia in past history. He underwent a cataract surgery, was admitted to the emergency department complaining of the gradual failing vision of the left eye within three months. The maximum corrected visual acuity was 20/40 OD and 20/100 OS. The pupils were still myotic despite the repeated attempts to make them dilate using pharmaceuticals Describe briefly 3 most probable diagnoses. Only the titles of clinical diagnoses	1. Secondary cataract (posterior capsule fibrosis) 2. Papillary block (glaucoma) 3. Microphakia syndrome with secondary complications
	***OCT + past history*** You are an ophthalmologist. Clinical case study: an 11-year-old boy with aphakia and microspherophakia in past history. He underwent a cataract surgery, was admitted to the emergency department complaining of gradual failing vision of the left eye within three months. The maximum corrected visual acuity was 20/40 OD and 20/100 OS. The pupils were still myotic despite the repeated attempts to make them dilate using pharmaceuticals. IRF, SRF were revealed on an OCT image of the macular area Describe briefly 3 most probable diagnoses. Only the titles of clinical diagnoses	1. Macular edema 2. **Retinal detachment (serous or exudative)** 3. Chronic uveitis with complications
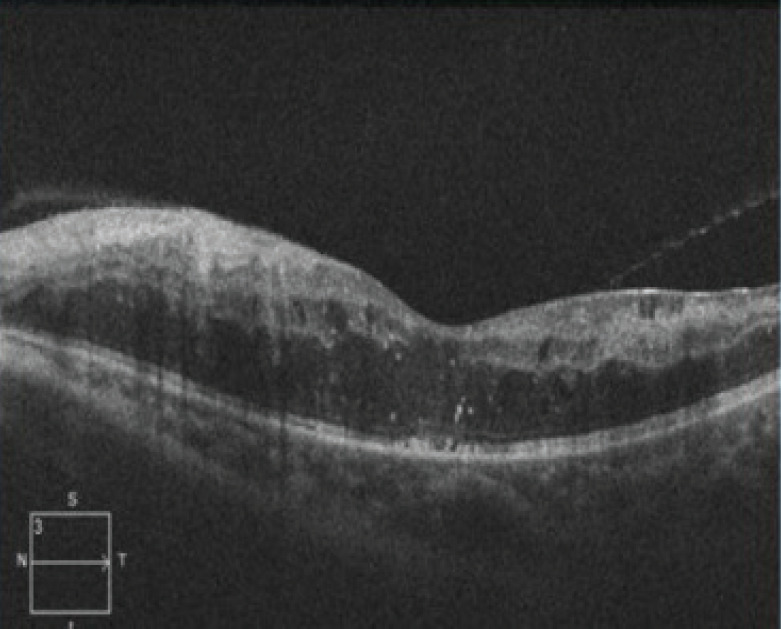 Verified diagnosis: **“radiation retinopathy”** The biomarkers labeled by the transformer model: **IRF, HE**	***OCT*** You are an ophthalmologist. Clinical case study: IRF, SRF were revealed on an OCT image of the macular area Describe briefly 3 most probable diagnoses. Only the titles of clinical diagnoses	1. Age-related macular degeneration 2. Central serous chorioretinopathy 3. Diabetic macular edema
	***Past history*** You are an ophthalmologist. Clinical case study: a 24-year-old man was referred for examination due to the painless decrease in vision clarity, more expressed in the right eye, lasting for about a month. In his past history there were the pineal gland tumor. The patient underwent craniospinal radiotherapy (36, 54, and 59.4 Gy). He had no accompanying diseases, such as diabetes or hypertension. On examination his visual acuity was 20/150 OD and 20/40 OS, and color perception — 0/12 and 7/14, respectively Describe briefly 3 most probable diagnoses. Only the titles of clinical diagnoses	1. **Radiation retinopathy** 2. Radiation optical neuropathy 3. Recurrent tumor of the pineal region with the optic pathway compression
	***OCT + past history*** You are an ophthalmologist. Clinical case study: a 24-year-old man was referred for examination due to the painless decrease in vision clarity, more expressed in the right eye, lasting for about a month. In his past history there were the pineal gland tumor. The patient underwent craniospinal radiotherapy (36, 54, and 59.4 Gy). He had no accompanying diseases, such as diabetes or hypertension. On examination his visual acuity was 20/150 OD and 20/40 OS, and color perception — 0/12 and 7/14, respectively. IRF, HE were revealed on an OCT image of the macular area Describe briefly 3 most probable diagnoses. Only the titles of clinical diagnoses	1. **Radiation retinopathy** 2. Chorioretinopathy (secondary, on radiotherapy) 3. Retinal vein occlusion (probably, caused by radiation)
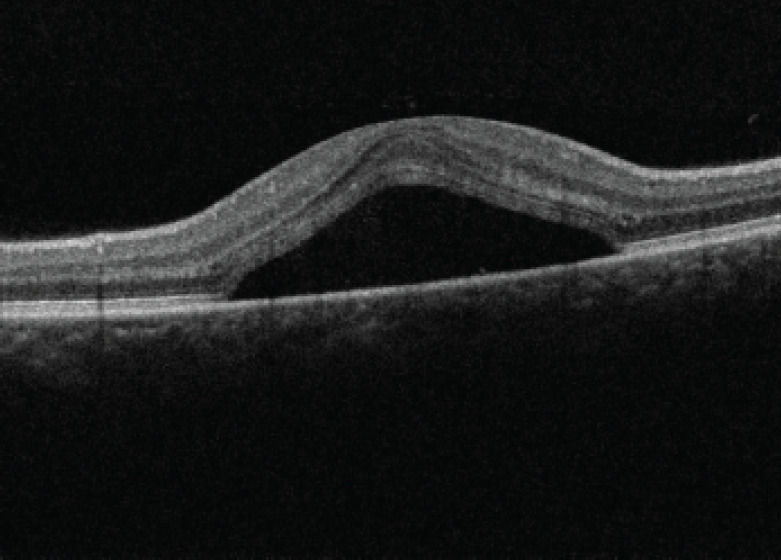 Verified diagnosis: “**central serous chorioretinopathy”** The biomarker labeled by the transformer model: **SRF**	***OCT*** You are an ophthalmologist. Clinical case study: SRF was revealed on an OCT image of the macular area Describe briefly 3 most probable diagnoses. Only the titles of clinical diagnoses	1. **Central serous chorioretinopathy** 2. Age-related macular degeneration 3. Choroidal neovascularization
	***Past history*** You are an ophthalmologist. Clinical case study: a 27-year-old man, an athlete, referred for treatment complaining of the blurred vision on the right eye, stress-associated, in his opinion Describe briefly 3 most probable diagnoses. Only the titles of clinical diagnoses	1. Myopia (nearsightedness) 2. Keratoconus 3. Retinal detachment
	***OCT + past history*** You are an ophthalmologist. Clinical case study: a 27-year-old man, an athlete, referred for treatment complaining of the blurred vision on the right eye, stress-associated, in his opinion. SRF was revealed on an OCT image of the macular area Describe briefly 3 most probable diagnoses. Only the titles of clinical diagnoses	1. **Central serous chorioretinopathy** 2. Age-related macular degeneration (rarely at the age of 27, although it is likely to occur in early forms of the disease) 3. Choroidal neovascularization (idiopathic or against the background of myopia)

## Discussion

The present study differs from the previously published works [14–16], since it suggested the approach to the multiclass classification of eight biomarkers on retinal OCT images based on combining ViT-Tiny-Patch16-224 architecture and current optimization methods. In contrast to the popular studies [17, 18, 20, 22] targeting primarily at segmentation, a binary classification or significantly larger architectures, the suggested technique demonstrates a compact model: Vision Transformer to be able to provide precision accuracy in a limited data volume and the great number of diagnostic categories.

In the course of the experiments, the model — ViT-Tiny-Patch16-224 — achieved the following values: Recall — 83.3%, Precision — 86.2%, and Specificity — 94.0%, it was comparable with the findings of other models. By comparison: Salehi et al. [18] used the model ResNet50 for the classification of eight classes of disease biomarkers on retinal OCT images, and the values were the following: Recall — 86.8% and Specificity — 87.4%. Katalevskaya et al. [17] used EfficientNetB0 combined with FPN for the segmentation of six biomarkers of the diseases on retinal OCT images, and the matric values of Recall and Specificity ranged 82.64–94.44% and 89.73–99.41%, respectively.

The replicate experiment involving 50 clinical ophthalmological cases and using the combination ViT-Tiny + DeepSeek showed the following values: Top-1 — 78%, Top-3 — 94%, and MRR — 84%. By comparison: the study by Zhou et al. [7] (150 dermatological cases) demonstrated the model SkinGPT-4 (ViT + GPT-4.0) to provide 80.63% of relevant answers and 75% of complete agreement of experts. And the study by Bahir et al. [4], who tested Gemini Advanced and ChatGPT-4 on 600 ophthalmological cases, showed the accuracy of the models to be 66 and 62%, respectively.

The suggested approach has a number of advantages: the use of a compact model reduces the computing costs, the integration of Vision Transformer with large language models demonstrates the prospects of the approach for medical diagnostics, while the architecture remains flexible and scalable that enables to adapt it to other tasks of an image analysis.

It should be noted that the small volume of the sampling and the limited number of biomarkers reduce the generalization ability of the model, and the differences in techniques and metrics between the studies under comparison prevent from making rigorous quantitative comparisons.

From a practical point of view, the developed approach can be applied as an accessory tool for the computer-aided analysis of OCT images putting forward primary diagnostics. However, for its complete implementation, further studies are needed on extended and standardized datasets including multi-center samplings.

## Conclusion

The present study succeeded in improving the differential diagnosis quality of retinal diseases in real clinical settings due to the suggested combined approach. The model ViT-Tiny-Patch16-224 (F1 macro — 0.84±0.03) showed the best results for a multiclass classification. On its basis we developed the interaction integration algorithm with a large language model DeepSeek-V3, which uses a sequential analysis of textual medical history data and imaging information on biomarkers on OCT images. Such combined architecture demonstrated the maximum efficiency achieving the following metric values: Top-1 Accuracy — 78%, Top-3 Accuracy — 94%, and MRR — 84%. The application of the approach enabled to improve the classification accuracy characteristics by more than 10% compared to the methods based on past history only; and by 50, 42 and 44% by the metrics: Top-1, Top-3, and MRR, respectively, compared to the methods using OCT biomarkers only.

The directions for further research include the expanded list of the biomarkers detected and the validation set increase due to the inclusion of rare, including orphan, retinal diseases.
